# Identification of the Marine Alkaloid Lepadin A as Potential Inducer of Immunogenic Cell Death

**DOI:** 10.3390/biom12020246

**Published:** 2022-02-02

**Authors:** Genoveffa Nuzzo, Carmela Gallo, Fabio Crocetta, Lucia Romano, Giusi Barra, Giuseppina Senese, Mario dell’Isola, Dalila Carbone, Valentina Tanduo, Federica Albiani, Guido Villani, Giuliana d’Ippolito, Emiliano Manzo, Angelo Fontana

**Affiliations:** 1 Bio-Organic Chemistry Unit, Institute of Biomolecular Chemistry, Consiglio Nazionale delle Ricerche, Via Campi Flegrei 34, Pozzuoli, 80078 Naples, Italy; carmen.gallo@icb.cnr.it (C.G.); lr.romanolucia@gmail.com (L.R.); giusi_barra@hotmail.it (G.B.); giusi.senese@gmail.com (G.S.); mario.service@libero.it (M.d.); d.car@hotmail.it (D.C.); fedealbiani@gmail.com (F.A.); gvillani@icb.cnr.it (G.V.); gdippolito@icb.cnr.it (G.d.); emanzo@icb.cnr.it (E.M.); 2 Department of Integrative Marine Ecology, National Institute of Biology, Ecology and Marine Biotechnology, Stazione Zoologica Anton Dohrn, 80121 Naples, Italy; fabio.crocetta@szn.it (F.C.); valentina.tanduo@szn.it (V.T.); 3 Laboratory of Bio-Organic Chemistry and Chemical Biology, Department of Biology, University of Naples “Federico II”, Via Cupa Nuova Cinthia 21, 80126 Naples, Italy

**Keywords:** marine natural products, drug discovery platform, lepadin A, antitumor effects, dendritic cells, immune system, immunogenic cancer cell death

## Abstract

Natural products and their synthetic analogs and derivatives are a traditional source of bioactive molecules with potential development as drug candidates. In this context, Marine Natural Products (MNPs) represent a rich reservoir of diverse molecular skeletons with potential pharmacological activity that, so far, has been mostly explored in cancer and infectious diseases. Starting from the development of a novel bioassay-guided screening platform for immunomodulatory compounds from an in-house MNPs library, we report the identification of the alkaloid lepadin A as a new model compound for immune-based anticancer activity with characteristics that suggest a possible mechanism as Immunogenic Cell Death inducer. The work describes the molecular-based bioprospecting in the Gulf of Naples together with the bioassay-guided fractionation, the chemical characterization of the alkaloid, and the biological activity in mouse dendritic cells (D1).

## 1. Introduction

The marine environment with its boundless chemodiversity represents a huge source of molecules with relevant potential in several pharmacological fields [1,2]. Among approved drugs based on compounds of marine origin, six are used in cancer therapy: cytarabine (Ara-C), eribulin mesylate (E7389), trabectedin (ET-743), brentuximab vedotin (SGN-35), polatuzamab vedotin and aplidin [3]. Many other compounds are currently under clinical investigations with promising anticancer activities [4].

Conventional anticancer drugs are cytotoxic substances having severe deleterious effects not only on tumor cells but also on normal cells, especially if they are in rapid replication. On the contrary, cancer immunotherapy uses the natural endowment of the immune system to prevent, control, and eliminate neoplastic cells. In theory, the resulting anticancer activity avoids side effects on physiological functions, which rapidly made training of the immune system to fight cancer a new therapeutic approach. A major breakthrough in this field was the introduction of checkpoint inhibitors, blocking antibodies of immune inhibitory receptors or molecular ligands, that have revolutionized the treatment of fatal malignancies [5]. In the last decade, research has increased the number of patients eligible for these treatments, introducing novel combinations of drugs and new druggable targets. This has broadened the types of immune-based cancer treatments, including cancer vaccines with prophylactic or therapeutic activity [6,7].

Despite these remarkable advances, cancer immune evasion and immune escape mechanisms are still major open issues in the design of effective anticancer immunotherapeutic strategies [8]. Not all tumors are immunogenic and, while many aggressive tumors are recognized by innate immune cells, the resulting immune response can be tuned down within the tumor microenvironment by different processes, including the production of numerous immunosuppressive molecules and the downregulation of the major histocompatibility complex (MHC) [9]. Thus, a major challenge has been the development of approaches to counteract these mechanisms, especially those linked to antigen presentation to T cells, and to improve immune surveillance.

In addition to the use of adjuvants, tumor immunogenicity can be increased by cell death that induces exposure or secretion of damage-associated molecular patterns (DAMPs) [10,11]. Immunogenic cell death (ICD) represents an emergent concept of functionally unique immune response to a cell death modality that comprises the induction of organellar and cellular stress and passive release of numerous DAMPs. Key elements in the immunogenic transduction of these signals are Dendritic Cells (DCs) and other Antigen Presenting Cells (APCs) that can sense DAMPs, by specific Pattern Recognition Receptors (PRRs) expressed on their membranes, and initiate a cellular cascade leading to activation of innate and adaptive immune responses [12]. This model was first proposed as an anticancer mechanism on the basis of clinical studies with conventional cytotoxic drugs [13] but, in recent years, other ICD inducers, including a few FDA-approved chemotherapeutics (e.g., anthracyclines, bortezomib), and stimulatory strategies have been reported [14,15]. Furthermore, direct effects on immune cells, including the activation of macrophages and DCs, also have been reported with several anticancer drugs. In such cases as well, there is a consensus that the ability to elicit innate immune response dictates the quality and efficacy of anticancer products.

With the aim of identifying adjuvant and immune-based anticancer candidates from natural extracts, we have recently implemented a screening platform that incorporates cytotoxic and immunomodulatory assays [16]. Here we report the identification of the alkaloid lepadin A from *Clavelina lepadiformis* sp. B (see below), already known to exhibit significant in vitro cytotoxicity against human cancer cell lines [17], as a potent activator of innate immune cells. The selection of the marine alkaloid was pursued by a novel procedure of bioassay-guided fractionation based on mouse DCs, a panel of nice tumor cell lines, and two orthogonal solid phase extraction (SPE) steps. Structural identification of the active molecule was carried out by using Nuclear Magnetic Resonance (NMR) and Mass Spectrometry (MS) techniques.

## 2. Materials and Methods

### 2.1. Sampling and Molecular Identification

Colonial ascidians of the *Clavelina lepadiformis* (Müller, 1776) species complex were collected on 9 June 2020, from the dock walls of the Fusaro Lake channel (Bacoli, Tyrrhenian Sea, Mediterranean Sea, 40.8229 N, 14.0498 E), from the surface to about 2 m depth. Samples were scraped from hard substrates with underwater knives, placed in single plastic bags filled with seawater, and brought alive to the laboratories of the Stazione Zoologica Anton Dohrn of Naples (SZN), Italy. Once there, samples were re-examined with the help of a Zeiss Axio Zoom.V16 microscope (Jena, Germany) to confirm putative conspecificity and then placed in buckets with filtered seawater and cleaned from possible contaminants. Finally, five single zooids were randomly isolated from the different colonies, fixed in 99.9% ethanol, and deposited in the collection of the Laboratory of Benthos (SZN-B-1046ASC15A, SZN-B-1048ASC15C–1051ASC15F) for subsequent taxonomic analyses. The remaining material was frozen for research of bioactive compounds.

The correct taxonomic identity of the colonies was further investigated through DNA barcoding and Bayesian Inference, as previous genetic data already showed that *C. lepadiformis* is composed in the Mediterranean Sea by two, almost indistinguishable, sibling species [18]. To do so, total genomic DNA was extracted from the ethanol-fixed zooids using the DNeasy Blood & Tissue kit (QIAGEN, Valencia, CA, USA), following the protocol used in Crocetta et al. [19]. Partial sequences of the *Cytochrome c Oxidase subunit I* (COI) gene were amplified from each DNA sample using the following primers designed by Tarjuelo et al. [20]: 5′-GTACTGAGCTTTCACAAACTGGGCAAT-3′ (forward) and 5′-TGAAAAAGAATAGGATCTCTCCTTCC-3′ (reverse). Polymerase chain reactions (PCRs) were conducted in 25 µL volume reaction as in Tanduo et al. [21]. Amplification was performed with an initial denaturation at 95 °C (5 min), followed by 39 cycles of denaturation at 95 °C (1 min), annealing at 47 °C (1 min), extension at 72 °C (1 min), with a final extension at 72 °C (5 min). The PCR products were purified and Sanger sequenced as in Tanduo et al. [22]. The chromatograms for each sequence obtained were checked, assembled, edited using BioEdit version 7.0.0, and compared with reference sequences from the NCBI nucleotide (NT) database using the Basic Local Alignment Search Tool (BLAST; www.ncbi.nih.gov/BLAST/, accessed on 1 December 2021) [23].

The phylogenetic analysis was constructed using the obtained COI fragments. Moreover, the NCBI data mining revealed the presence of 27 COI partial sequences of *C. lepadiformis*, that were downloaded together with two sequences of *C. oblonga* Herdman, 1880 and one sequence of *Didemnum vexillum* Kott, 2002 to be used as outgroup, based on Reinhardt et al. [24]. Sequences were aligned using ClustalW (2.1) on the CIPRES Science Gateway [25], using default parameters. Identical sequences were deleted after and before trimming. The evolutionary model was selected through the AICc (corrected Akaike Information Criterion) algorithm, implemented in JModelTest 2 v.0.1.10 [26]. Bayesian Inference (B.I.) was performed using MrBayes v.3.2.5 for 10 million generations, a sampling interval every 1000 generations, and discarding 25% of the produced trees [27]. Tracer v1.7.1 [28] was used to check the convergence of MCMC runs. The tree obtained was checked by eye in FigTree 1.3.1 and edited in Inkscape 0.92.

### 2.2. Extraction and HRX-SPE Fractionation

The ascidian samples (150 g wet weight) were lyophilized to give 17 g of brown powder (dry weight). About 6 g this material was extracted with methanol (Merk Life Science S.r.l., Milan, Italy) using a tissue homogenizer Precellys Evolution equipped with a cooling system Cryolys Evolution (Bertin Italia, Genoa, Italy), to obtain 600 mg of crude extracts. This protocol of extraction consisted of a run at 6200 rpm (3 cycles × 30 s), at the temperature of 16 °C to prevent degradation, followed by centrifugation of the sample at 3450 rpm for 10 min at 4 °C. The extract was filtered with a rinsed filter paper and dried in a rotatory evaporator using a maximum temperature of 24 °C. This full crude extract was weighted and aliquoted using methanol-dichloromethane 2:1 (*v*/*v*). Finally, it was dried under a nitrogen flow and kept at −80 °C until further use.

About 60 mg of raw extract was subjected to SPE on a GX-271 ASPEC Gilson apparatus (Gilson Italy, Cinisello, Italy) by using CHROMABOND^®^ HRX cartridges (6 mL/500 mg, Macherey-Nagel, Düren, Germany) as reported by Cutignano et al. [29]. This extraction yielded five fractions (A, B, C, D and E) eluted with H_2_O, CH_3_OH/H_2_O 5:5, CH_3_CN/H_2_O 7:3, CH_3_CN, and CH_2_Cl_2_/CH_3_OH 9:1, respectively. Both raw extract and SPE fractions B–E were tested. Fraction A mainly composed of sea salt was not further analyzed. The distribution of metabolites in the enriched SPE fractions were monitored by thin layer chromatography (TLC) (Ce(SO_4_)_2_ was used as staining) and ^1^H NMR.

### 2.3. HILIC-SPE Fractionation

SPE-HRX fraction C (6 mg) was subjected to a successive solid-phase extraction using a hydrophilic interaction chromatography (or hydrophilic interaction liquid chromatography, HILIC), using a prepacked column CHROMABOND^®^ HILIC cartridges (6 mL/500 mg, Macherey-Nagel, Düren, Germany) and the automated GX-271 ASPEC Gilson system. In detail, the cartridge was conditioned with 2 mL of milli-q water and equilibrated with 10 mL of tetrahydrofuran (THF)/*n*-hexan 50:50 (*v*/*v*). The sample was suspended in 1 mL of THF/*n*-hexan 50:50 (*v*/*v*) and sonicated for few seconds in an ultrasonic bath before loading onto the column. Elution steps are reported in Table 1. The distribution of the metabolites were analyzed by TLC stained with Ce(SO_4_)_2_. Furthermore, NMR and MS spectra analysis were recorded for dereplication purpose.

### 2.4. Purification and Characterization of Lepadin A

Raw extract (370 mg) from the *C. lepadiformis* species complex was fractionated by sephadex LH-20, using methanol as eluent, to obtain 49 mg of a crude sample containing lepadin A. Further purification on silica column starting with a gradient of petroleum ether/diethyl ether followed with chloroform and chloroform/methanol gradient led to the elution of lepadin A (2.8 mg) in CHCl_3_/MeOH 9:1 (*v*/*v*). Pure compound was achieved by HPLC on Luna NH2 (250 × 4.6 mm, 5 µm) in isocratic condition with a mixture of isopropanol/*n*-hexan 5:95 (*v*/*v*) and 1% of NH_4_OH at a flow of 1 mL/min. NMR data (Figures S3–S6 and Table 2) were recorded in CD_3_OD and CD_3_OD/CDCl_3_ 1:1 (*v*/*v*) to compare with literature reference [30]. High resolution EI-MS confirmed the molecular formula C_20_H_34_NO_3_ ([MH]^+^, *m*/*z* = 336.2462; calculated for [MH]^+^ 336.2533).

### 2.5. Biological Assay

#### 2.5.1. Cytotoxicity on Cancer Cell Lines

Cytotoxicity was assessed on the following tumor cell lines: CALU-1, CALU-3, HCC827, MALME-3M, A375, A2058, KMS-12, RPMI 8226, JJN-3, cultured as reported on Gallo et al. [16]. Each tumor cell line was cultured at the concentration of 1 × 10^4^ in 0.1 mL of medium, in a 96 well plate. The organic fractions were diluted at maximum concentration of 3 mg/mL in DMSO and tested at 5 and 30 µg/mL or 2.5 and 10 µg/mL for HRX and HILIC samples, respectively. Cells with 1% DMSO in 0.1 mL of medium were used as blank. As positive controls Cisplatin, MEK inhibitor, and doxorubicin were all used at the concentration of 100 µM. All conditions were plated in duplicate and cells were incubated for 24 h. For cell lines growing in adherence, the Sulforodamine B (SRB) Assay Kit (Abcam ab235935, Milan, Italy) was performed. After 24 h of treatment, cells were fixed by a fixation solution for 1 h. After 3 washes in H_2_O, cells were stained with SRB solution for 15 min and rinsed with washing solution for 4 times. Protein-bound dye was solubilized, and the optical density was determined at 545 nm according to manufacturer instructions. MTS Proliferation Assay Kit (Abcam, ab197010, Milan, Italy) was used for cells growing in suspension. 10 µL MTS (3-(4,5-dimethylthiazol-2-yl)-5-(3-carboxymethoxyphenyl)-2-(4-sulfophenyl)-2H-tetrazolium) was added to each well and incubated at 37 °C for 4 h. The absorbance was measured at 490 nm. For all the experiments, percent of cytotoxicity was calculated as: [(O.D. vehicle) × (O.D. sample)/O.D. vehicle] × 100. Background correction was carried out by subtracting the O.D. of culture media.

#### 2.5.2. D1 Cell Assay

D1 cells were maintained in IMDM supplemented with 30% and then in 15% R1-conditioned medium as described in Gallo et al. [16]. These cells were plated on an untreated white flat 96-well plate at a density of 1.5 × 10^4^ cell in 0.2 mL complete culture medium and incubated for 24 h after treatment. Compounds were dissolved in MeOH at the maximum concentration of 0.3 mg/mL. Of this solution, 0.05 mL were used to perform the coating of the plate. After 24 h, plates were centrifuged at 300× *g* for 3 min and washed with staining buffer (SB) (2% FBS; 0.1% sodium azide in PBS). Staining was performed with monoclonal antibody anti MHC-II APC, CD80 FITC, CD40 PE (REA custom mix from Miltenyi Biotech, Auburn, CA, USA). Before acquisition, each sample was incubated with Propidium iodide solution (Invitrogen, Thermo Fisher Scientific, Waltham, MA, USA) for 5 min at room temperature.

#### 2.5.3. Statistical Analysis

All data were analyzed by one-way ANOVA followed by the Tukey test for a multiple comparison test. A *p*-value less than 0.05 was considered as statistically significant. All analyses were performed using the GraphPad Prism 8.00 for Windows software (GraphPad Software, San Diego, CA, USA). The EC_50_ value was calculated by Non Linear regression analysis and EC_50_ shift function using the GraphPad Prism software.3.

## 3. Results and Discussion

### 3.1. Species Collection and Identification

Based on their morphology, all colonies resulted as belonging to the *C. lepadiformis* species complex. In particular, the colonies were formed by zooids (10–30) of about ~1–2 cm in height with stolons not fused among them. A 520 base pairs (bp) partial sequence of the COI gene was obtained from the five colonies, with all sequences resulting identical to each other. A single sequence was thus deposited in GenBank (OM278387). It showed a 98.10–100% similarity with 19 sequences deposited as *C. lepadiformis*, but also a 95.08–95.90% similarity with eight additional sequences deposited under the same binomial name (Table S1). After the first alignment, six identical sequences were deleted from the dataset (HM012483, HM012482, and four from the Fusaro Lake), and the same was done with three additional sequences (FJ839918, AY603104, and AM292603) that resulted identical after trimming.

**Figure 1:**
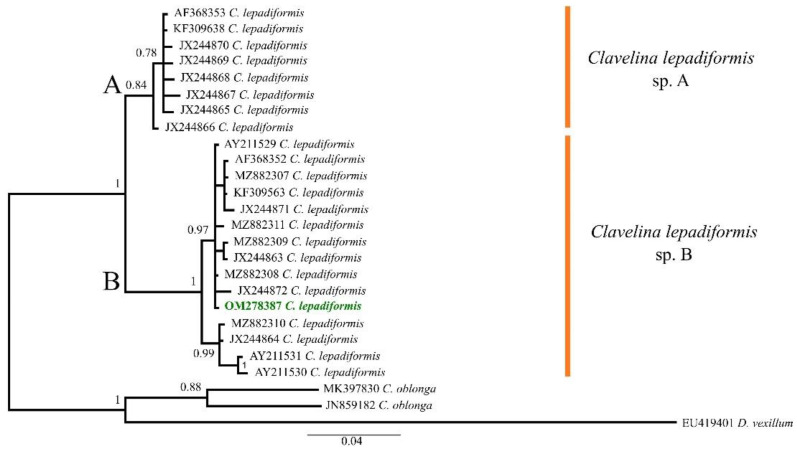
Bayesian Inference tree of the *Clavelina lepadiformis* species complex based on COI partial sequences downloaded from GenBank (accession codes as in Table S1) and the one from the Fusaro Lake (Bacoli, Tyrrhenian Sea, Mediterranean Sea) highlighted in green bold. Numbers above/below branches represent posterior probabilities. Scale bar represents nucleotide substitution.

Overall, 23 sequences of *C. lepadiformis* were used to infer the phylogenetic analysis (Table S2), with a final alignment that consisted of 26 sequences of 425 bp. The selected model was the HKY + I. The B.I. (−*lnL* = 1473.60 for run 1; −*lnL* = 1470.33 for run 2) produced a tree that divided *C. lepadiformis* in two different clades, hereafter named *C. lepadiformis* sp. A and *C. lepadiformis* sp. B (B.I. = 1) (Figure 1).The species A (B.I. = 0.84) mostly included samples collected from open rocky seashores and previously ascribed to the “Mediterranean exterior” clade *sensu* Turon et al. [18], although they also clustered together with the sequence KF309638, obtained from a colony collected from a Mediterranean harbour by López-Legentil et al. [31] and thus potentially falling in the “Mediterranean interior and Atlantic” clade *sensu* Turon et al. [18]. Nonetheless, the species B (B.I. = 1) was only formed by specimens ascribed to the “Mediterranean interior and Atlantic”, that clustered well together with our samples. Therefore, the species worked here was molecularly identified as *C. lepadiformis* sp. B.

### 3.2. Screening Platform and Bioassay-Guided Fractionation

In recent years, we have progressively implemented a bioassay-guided screening platform starting from a pre-fractionation of crude extracts by Solid Phase Extraction (SPE) on a spherical, hydrophobic polystyrene-divinylbenzene resin that allows simple loading of the crude material in water, desalting and enrichment of the active components in chemically homogeneous fractions [29]. This approach led to the discovery of a novel class of sulfoglycolipid adjuvants, collectively named Sulfavants, with unconventional immunomodulatory activity on DCs and in vivo antigen-specific immunization [32]. Recently, the technology has been further tested on a library of extracts for cytotoxic, antibiotic and anti-diabetic activity [33].

**Figure 2:**
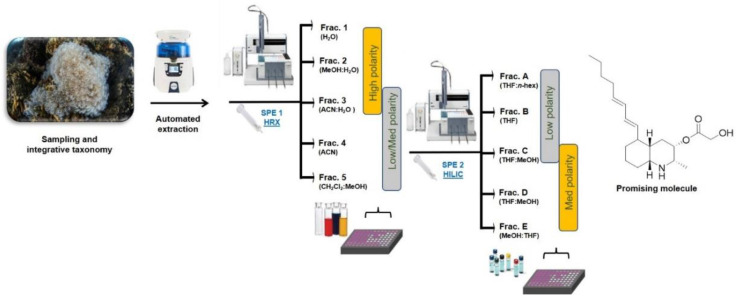
Schematic view of the bioassay-guided fractionation platform used for the screening of natural small molecules with immune-based anticancer activity from marine extracts. SPE-HRX = Solid Phase Extraction-Hydrophobic Polystyrene-Divinylbenzene; SPE-HILIC = Solid Phase Extraction-Hydrophilic Interaction Liquid Chromatography.

In the present study, the screening platform has been further improved with a second fractionation step based on an SPE protocol using hydrophilic interaction liquid chromatography (HILIC) (Figure 2). This step allows an effective separation of small polar compounds, thus providing a chromatographic resolution that is based on chemical interactions that are opposite to the hydrophobicity-driven fractionation achieved by polystyrene-divinylbenzene resin. The combination of the sequential hydrophobic and hydrophilic phases determines an orthogonal chromatographic approach that allows quick dereplication of complex mixtures of small scale. The protocol has been developed on prepacked HILIC cartridges using the automated GX-271 ASPEC Gilson system and it has set up to obtain five fractions (A–E) (Table 1) with an average recovery of about 95% of the organic compounds loaded on the column.

The mouse immature dendritic cells derived from mouse spleen (D1 cells) used in this study were previously described and it was demonstrated that their maturation can be combined with tests of viability on tumor cell lines in order to select immune-based anticancer molecules [16]. Tumor cells and D1 cells were treated with two concentrations of crude extract or SPE fractions and activity was reported by heatmaps showing the results in comparison to the lipopolysaccharide (LPS), as positive control for D1 cell activation [34], and MEK inhibitor, cisplatin and doxorubicin as positive controls for anticancer and ICD activity [14].

Transition from an “immature” to a “mature” status of D1 cells was detected by the quantitative assessment of the histocompatibility complex II (MHC-II) and costimulatory molecules CD80 and CD40 on the cell membranes by flow cytometry. These factors are functional for an efficient priming of T lymphocytes by DCs and provides diagnostic probes of the activation of the adaptive immune system and the generation of protective antitumor immunity [35,36].

### 3.3. Selection of Lepadin A as ICD Inducer

As part of a growing biological collection currently composed of more than 150 marine organisms, samples of *C. lepadiformis* sp. B were lyophilized (dry weight 17 g) prior to be extracted with the organic solvent (methanol). Only 6 g of the organic material was extracted for the screening, using a tissue homogenizer equipped with a cooling system, to obtain an exhaustive recovery of metabolites. This crude extract was weighted and aliquoted before the first SPE-HRX fractionation. A small amount (about 60 mg) of raw extract was subjected to the hydrophobic SPE as reported by Cutignano et al. [29].

**Figure 3:**
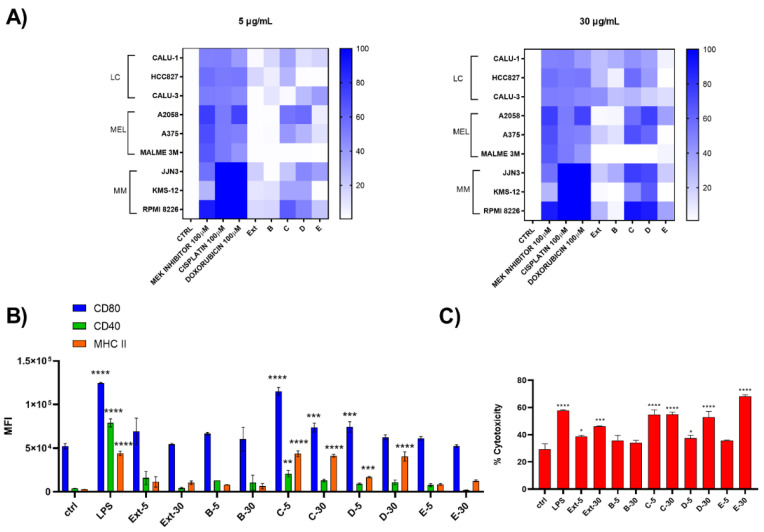
Biological screening of HRX fractions of the extract of *C. lepadiformis* sp. B. (**A**) Heat map of cytotoxicity assays carried out on the panel of nine different cell lines treated at 5 and 30 ug/mL with positive controls, total extract (Ext) and HRX-SPE fractions (B–E) of *C. lepadiformis* sp. B. Values reported in the color bar legend on the right indicate the % of cytotoxicity; (**B**) Surface expression analysis of CD80, CD40, and MHC-II on D1 treated at 5 and 30 ug/mL extract (Ext) and HRX-SPE fractions (B–E) of *C. lepadiformis* sp. B. All data were compared to the cells treated with either the vehicle (Ctrl) or LPS (positive control). Data are expressed as MFI (mean fluorescence intensity) measured for each marker; (**C**) Percentage of cytotoxicity of *C. lepadiformis* sp. B extract and fractions at the same concentrations on D1 cells. After 24 h, treated and untreated cells were stained by iodide propidium. Statistical significance (* *p* < 0.5; ** *p* < 0.01; *** *p* < 0.001, **** *p* < 0.0001) was established by non-parametric two-sample Wilcoxon Test (two-side alternative).

Two different concentrations (5 and 30 μg/mL) of both raw extract and SPE-derived enriched fractions were tested on chronic forms of lung carcinoma (LC), melanoma (Mel), and multiple myeloma (MM) and on D1 cells (Figure 3). Results showed that HRX fraction C and D were active both at 5 and 30 µg/mL with an enrichment of the activity compared to the total extract, mostly noticeable on Mel and MM cancer cell lines. In particular on melanoma cells, these fractions were inactive on MALME but were cytotoxic on the other two tested cell lines, whereas, regarding multiple myeloma cells, RPMI8226 was the most sensitive to the treatment (Figure 3A). HRX fraction C (6 mg) was also found able to induce the maturation of D1 cells (Figure 3B), as indicated by the significant increase in the expression of MHC-II and the costimulatory molecules CD80 and CD40, with a mild toxicity on these cells at both tested concentrations (Figure 3C).

The HRX active fraction C was then subjected to HILIC chromatography according to the design of the screening platform described above (Figure 2). Biological assays on the five fractions (A–E) obtained by this second fractionation confirmed the immunomodulatory activity on D1 cells, with an enrichment of the activity especially on the new fraction C that was active at the non-cytotoxic concentration of 2.5 μg/mL (Supplementary Figure S1). Preliminary dereplication analysis by ^1^H NMR of the active HILIC fraction C (Figure S2) clearly showed the presence of a predominant metabolite.

### 3.4. Validation of the Biological Activity

**Figure 4:**
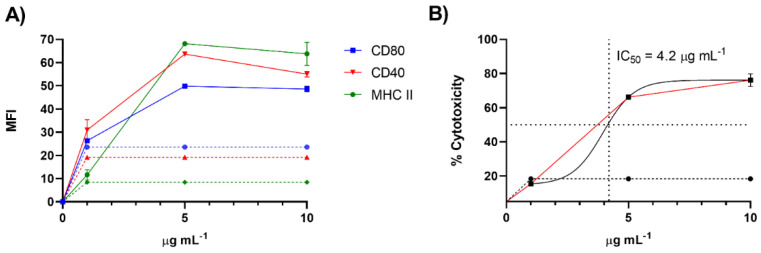
Maturation and toxicity on D1 cells induced by the active product purified from the extract of *C. lepadiformis* sp. B. (**A**) Expression of cell surface markers of D1 cells after treatment with the active molecule from 1 to 10 µg/mL (n = 3). Data are expressed as Mean Fluorescence Intensity (MFI) of CD80, CD40 and MHC-II compared to cells treated only with vehicle (dashed lines) after 24 h; (**B**) Cell viability expressed as percentage of cytotoxicity after stimulation of D1 cells with the active molecule in the same range of concentrations. A nonlinear regression analysis was performed for the estimation of the IC_50_ value as plotted in the figure.

To further validate our screening procedure, we purified the active molecules from 370 mg of raw extract through size exclusion chromatography (Sephadex LH-20) followed by silica gel column and HPLC fractionation (See Materials and Methods section). The isolated compound was tested on the immune cells at concentrations ranging from 1 to 20 μg/mL. As reported in Figure 4A, the tests on D1 cells confirmed the immunomodulatory activity with an EC_50_ of 1.64 ± 0.02 µg/mL. Toxic activity on the same cell line was at 4.20 ± 0.14 µg/mL (Figure 4B).

### 3.5. Structure Characterization of Lepadin A as Active Molecule in the Extract of “C. lepadiformis”

**Figure 5:**
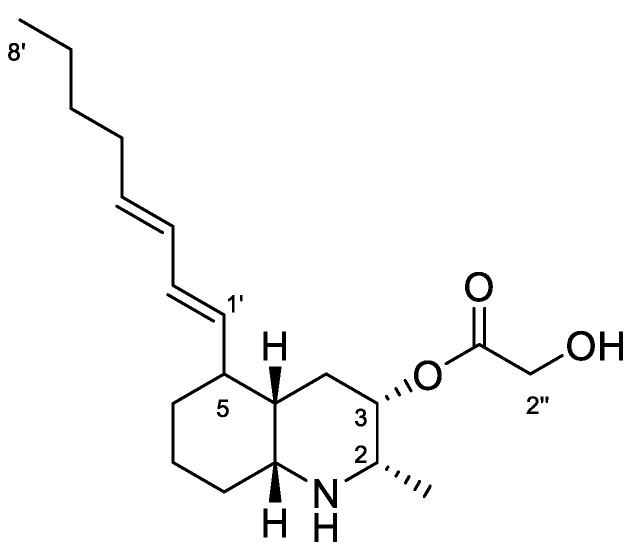
Chemical structure of Lepadin A (**1**).

Lepadin A (**1,** Figure 5) was first isolated in 1991 by Steffan [30] from specimens of the *C. lepadiformis* species complex collected in the North Sea, and considered the first decahydroquinoline alkaloid obtained from a marine natural source. Subsequently, the related compounds lepadins B and C, along with lepadin A, were reported from the flatworm *Prostheceraeus villatus* (Montagu, 1815) (incorrectly reported as *P. villatus*), which mainly feeds on clavelinid ascidians [17]. These compounds have been demonstrated to possess in vitro cytotoxicity on murine leukemia, human breast cancer, glioblastoma/astrocytoma, ovarian carcinoma, colon and lung [17]. More recently (2002), Wright et al. [37] reported the isolation of lepadins D–F from an unidentified tunicate of the genus *Didemnum* Savigny, 1816, and Davis et al. [38] reported lepadins F–H from the Australian ascidian *Aplidium tabascum* Kott, 1992. Lepadins D–F are described to possess significant and selective antiplasmodial and antitrypanosomal activity [37], whereas lepadin B was also found to block neuronal nicotinic acetylcholine receptors [39]. Recently, three new variants of the decahydroquinoline core differing by the presence of a rare 3-methylthioacrylate ester at C-3, lepadins I–K, were isolated from a *Didemnum* species collected in the Bahamas [40].

Lepadin alkaloids are characterized by a common structural framework of a 2,3,5-trisubstituted cis-decahydroquinoline ring containing a C-2 methyl group, a C-3 oxygenated (hydroxy or acyloxy) group, and a C-5 eight-carbon side chain. ^1^H NMR spectrum in CD_3_OD of the active fraction showed an AB system at 4.23 ppm ascribable to the butadienyl system, two methyl groups (a doublet at 1.10 ppm on C-2 and the triplet terminal at 0.92 ppm), a conjugated system of double bonds between 5.32–5.98 ppm. The down-shifted signals also included protons at δ 4.91, 4.23, 3.02 and 2.98 that were in agreement with the structure of lepadin A (Figure 5). Further spectroscopic experiment (Figures S3 and S4) and ESI^+^ MS analysis confirmed the depicted structure. Finally, ^1^H NMR data in CDCl_3_:CD_3_OD (Table 2 and Figure S5) were completely superimposable with those reported in the literature [30].

## 4. Conclusions

Conventional anticancer chemotherapy is generally related to killing or irreversibly arresting the growth of tumor cells. Consequently, cell death, both due to a physiological, regulated and non-immunogenic process whose prototype is apoptosis, or to a pathological, incontrollable cellular failure, such as necrosis, has been until now the main target in the search for anticancer candidates. However, chemotherapy agents can have a profound impact on the host immune system and although, to our knowledge, no systematic analysis of the immune-based effects of chemotherapeutic agents has been carried out so far, it is now becoming evident that these mechanisms can represent a key therapeutic aspect to the successful development of new drugs to fight cancer.

Lepadins are *cis*-fused decahydroquinoline (DHQ) marine alkaloids that have shown diverse biological activities and have attracted extensive synthetic interest [41]. In this study we found that lepadin A shows cytotoxic effect against cancer cells together with maturation of mouse DCs at micromolar concentrations. The combination of the two effects is expected to increase the anticancer properties by a synergistic mechanism deriving from both the role of mature DCs in the generation of anti-tumor activity and the release of immunogenic molecules by dying cells. Both mechanisms are potential approaches to breaking tumor evasion, thus suggesting that lepadin A could be a good candidate for further studies on immune-based anticancer activity. In this view, it is particularly noticeable that the marine alkaloid triggers a significant over-expression of MHC-II and co-stimulatory molecules that are key signals for naïve T cell differentiation by DCs and for mounting an effective immune response.

Priming of APCs, especially DCs, and cell death are two key characteristics of the immunogenic cell death. On this basis, lepadin A has the prerequisites of ICD inducer. To this aim, it is noteworthy that the immune response of **1** occurs at a concentration (EC_50_ = 1.64 ± 0.02 µg/mL) that is almost three times lower than that required to induce cell death (IC_50_ = 4.20 ± 0.14 µg/mL). This may indicate that at subtoxic concentrations the molecule can induce a cell stress that is another potential cause of ICD. Further studies to investigate the mechanism of action of lepadin A and validate the ICD induction are ongoing.

## Figures and Tables

**Table 1:** Elution protocol on Hydrophilic Interaction Liquid Chromatography-Solid Phase Extraction (HILIC-SPE) column (6 mL/500 mg) by using automated stepwise elution.

Sample Preparation	Column Activation	Elution Gradient
Add 1 mL of THF/*n*-hexane 50:50 (*v*/*v*) and sonicate.	2 mL H_2_O; 10 mL THF/*n*-hexane 50:50 (*v*/*v*).	THF/*n*-hexane 50:50 *v*/*v* (6 mL) THF 100% (6 mL) THF/MeOH 90:10 *v*/*v* (6 mL) THF/MeOH 80:20 *v*/*v* (6 mL) THF/MeOH 10:90 *v*/*v* (6 mL)

**Table 2:** ^13^C NMR (125 MHz) and ^1^H NMR (600 MHz) data for lepadin A in CD_3_OD ^a^ and CDCl_3_/CD_3_OD ^b^ 1:1 *v*/*v*.

Position	^13^C δ ^a^ (ppm), Type	^1^H δ ^a^ (ppm), Multiplicity (*J* in Hz)	^1^H δ ^b^ (ppm), Multiplicity
2	56.4, CH	2.98, app. q (6.0)	2.95, app. dq
3	72.4, CH	4.91, m	4.93, m
4	33.3, CH_2_	1.70, m 2.16, app. dt (15.0; 2.0)	1.69, m 2.18, app. dt
4a	39.5, CH	1.38, m	1.37, m
5	41.0, CH	2.57, m	2.50, m
6	35.3, CH_2_	1.12, m 1.66, m	1.14, m 1.67, m
7	21.4, CH_2_	1.58, m	1.58, m
8	32.8, CH_2_	1.85, m 1.65, m	1.81, m 1.67, m
8a	56.0, CH	3.02, app. s	3.00, app. s
1′	137.6, CH	5.32, dd (14.5; 8.5)	5.30, dd
2′	133.2, CH	6.04, dd (14.5; 10.0)	5.98, dd
3′	131.7, CH	5.98, dd (14.5; 10.0)	5.96, dd
4′	133.5, CH	5.59, dd (14.5; 7.0)	5.57, dd
5′	33.1, CH_2_	2.09, q (7.0)	2.05, q
6′	23.2, CH_2_	1.40, m	1.38, m
7′	32.1, CH_2_	1.37, m	1.36, m
8′	14.5, CH_3_	0.92, t (7.0)	0.90, t
1′′	n.d.		
2′′	61.6, CH_2_	4.23, AB system (16.5)	4.23, AB system
2-Me	17.8, CH_3_	1.10, d (6.5)	1.09, d

^a^ Chemical shifts of ^13^C and ^1^H are given in ppm relative to the solvent peak of CD_3_OD at 49.0 and 3.34 ppm respectively. ^b 1^H Chemical shifts are given in ppm relative to the solvent peak of CD_3_OD at 3.35 ppm as reported in Steffan [30]. n.d. = not determined.

## Supplementary Materials

The following are available online at https://www.mdpi.com/article/10.3390/biom12020246/s1, Figure S1: Surface expression analysis of CD80, CD40, and MHC-II, and percentage of vitality on D1 treated at 2.5 and 10 µg/mL with fraction C from the HRX-SPE and fractions A–E from the HILIC-SPE. All data were compared to cells treated with vehicle (Ctrl) or LPS (positive control). The color bar on the right shows the MFI (mean fluorescence intensity) measured for each marker, Figure S2: ^1^H NMR (600 MHz, in CD_3_OD) spectrum of the fraction C from HILIC-SPE, Figure S3: ^1^H NMR (600 MHz, CD_3_OD) spectrum of lepadin A from *C. lepadiformis* sp. B, Figure S4: ^1^H-^1^H COSY NMR (600 MHz, CD_3_OD) spectrum of Lepadin A from *C. lepadiformis* sp. B., Figure S5: HSQCed NMR (600 MHz, CD_3_OD) spectrum of lepadin A from *C. lepadiformis* sp. B, Figure S6: ^1^H NMR (600 MHz, CDCl_3_:CD_3_OD 1:1 *v*/*v*) spectrum of lepadin A from *C. lepadiformis* sp. B, Table S1: Blast percentage of similarity of “*Clavelina lepadiformis*” sequences from the Fusaro Lake (Bacoli, Tyrrhenian Sea, Mediterranean Sea) (sequences after Tarjuelo et al. [20], Turon et al. [18], Turon and López-Legentil [42], Gissi et al. [43], Reinhardt et al. [24], Stach et al. [44], Rius et al. [45], López-Legentil et al. [31], Holman et al. [46]), Table S2: GenBank COI partial sequences of *Clavelina* taxa (*C. lepadiformis* and *C. oblonga*) (after Blast results, Goddard-Dwyer et al. [47], and Rocha et al. [48]) and *Didemnum vexillum* (after Stefaniak et al. [49]) used in the phylogenetic analysis and associated specimen data (codes and clades/haplotypes after GenBank, Tarjuelo et al. [20], Turon et al. [18], and Rius et al. [45]; geographic localities obtained from GenBank and/or relevant paper/s). Abbreviations used: EAO—Eastern Atlantic Ocean; MED—Mediterranean Sea; PO—Pacific Ocean; SA—South Africa; WAO—Western Atlantic Ocean.
