# Supplementary material for: Identification of the Marine Alkaloid Lepadin A as Potential Inducer of Immunogenic Cell Death
Source: Biomolecules. 2022 Feb 2;12(2):246. doi: 10.3390/biom12020246 (PMC8961536; doi:10.3390/biom12020246)
Supplement: Supplementary file 1 [file biomolecules-12-00246-s001.zip › biomolecules-1560348-supplementary.pdf]

Supplementary Materials

Identification of the Marine Alkaloid Lepadin A as Potential Inducer of Immunogenic Cell Death.

Genoveffa Nuzzo, Carmela Gallo, Fabio Crocetta, Lucia Romano, Giusi Barra, Giuseppina Senese, Mario dell'Isola, Dalila Carbone, Valentina Tanduo, Federica Albiani, Guido Villani, Giuliana d'Ippolito, Emiliano Manzo and Angelo Fontana

Figure S1. Surface expression analysis of CD80, CD40, and MHC-II, and percentage of vitality on D1 treated at 2.5 and 10 μg/mL with fraction C from the HRX-SPE and fractions A–E from the HILIC-SPE. All data were compared to cells treated with vehicle (Ctrl) or LPS used as positive control. The color bar on the right shows the MFI (mean fluorescence intensity) measured for each marker.
Figure S2. ^{1}H NMR (600 MHz, in CD_3OD) spectrum of the fraction C from HILIC-SPE.
Figure S3. ^{1}H NMR (600 MHz, CD_3OD) spectrum of Lepadin A from *C. lepadiformis* sp. B.
Figure S4. ^{1}H-^{1}H COSY NMR (600 MHz, CD_3OD) spectrum of Lepadin A from *C. lepadiformis* sp. B.
Figure S5. HSQCed NMR (600 MHz, CD_3OD) spectrum of Lepadin A from *C. lepadiformis* sp. B.
Figure S6. ^{1}H NMR (600 MHz, $CDCl_3$:CD_3OD 1:1 *v*/*v*) spectrum of Lepadin A from *C. lepadiformis* sp. B.
Table S1. Blast percentage of similarity of "*Clavelina lepadiformis*" sequences from the Fusaro Lake (Bacoli, Tyrrhenian Sea, Mediterranean Sea) (sequences after Tarjuelo et al. [20], Turon et al. [18], Turon and López-Legentil [42], Gissi et al. [43], Reinhardt et al. [24], Stach et al. [44], Rius et al. [45], López-Legentil et al. [31], Holman et al. [46]).
Table S2. GenBank COI partial sequences of *Clavelina* taxa (*C. lepadiformis* and *C. oblonga*) (after Blast results, Goddard-Dwyer et al. [47], and Rocha et al. [48]) and *Didemnum vexillum* (after Stefaniak et al. [49]) used in the phylogenetic analysis and associated specimen data (codes and clades/haplotypes after GenBank, Tarjuelo et al. [20], Turon et al. [18], and Rius et al. [45]; geographic localities obtained from GenBank and/or relevant paper/s). Abbreviations used: EAO—Eastern Atlantic Ocean; MED—Mediterranean Sea; PO—Pacific Ocean; SA—South Africa; WAO—Western Atlantic Ocean.

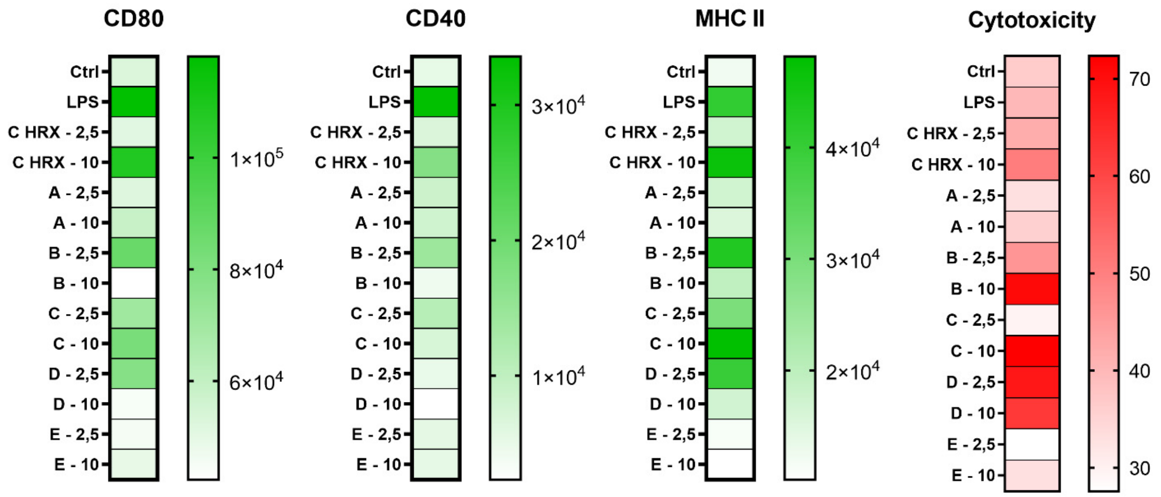

Figure S1. Surface expression analysis of CD80, CD40, and MHC-II, and percentage of vitality on D1 treated at 2.5 and 10 μg/mL with fraction C from the HRX-SPE and fractions A–E from the HILIC-SPE. All data were compared to cells treated with vehicle (Ctrl) or LPS used as positive control. The color bar on the right shows the MFI (mean fluorescence intensity) measured for each marker.

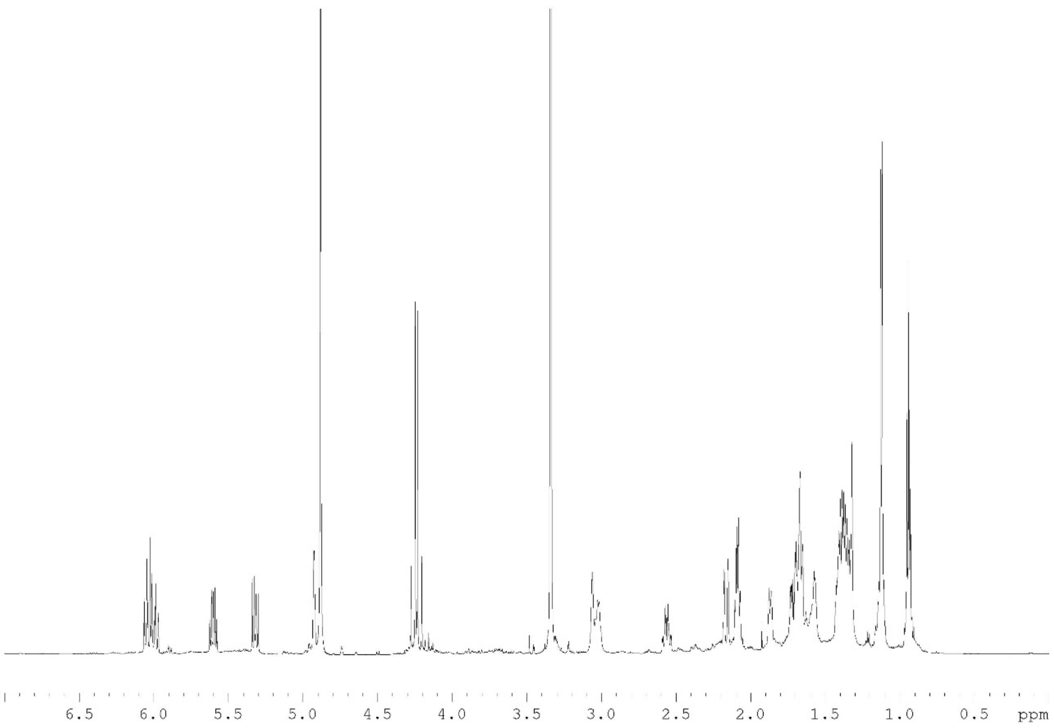

Figure S2. ^{1}H NMR (600 MHz, in CD_3OD) spectrum of the fraction C from HILIC-SPE.

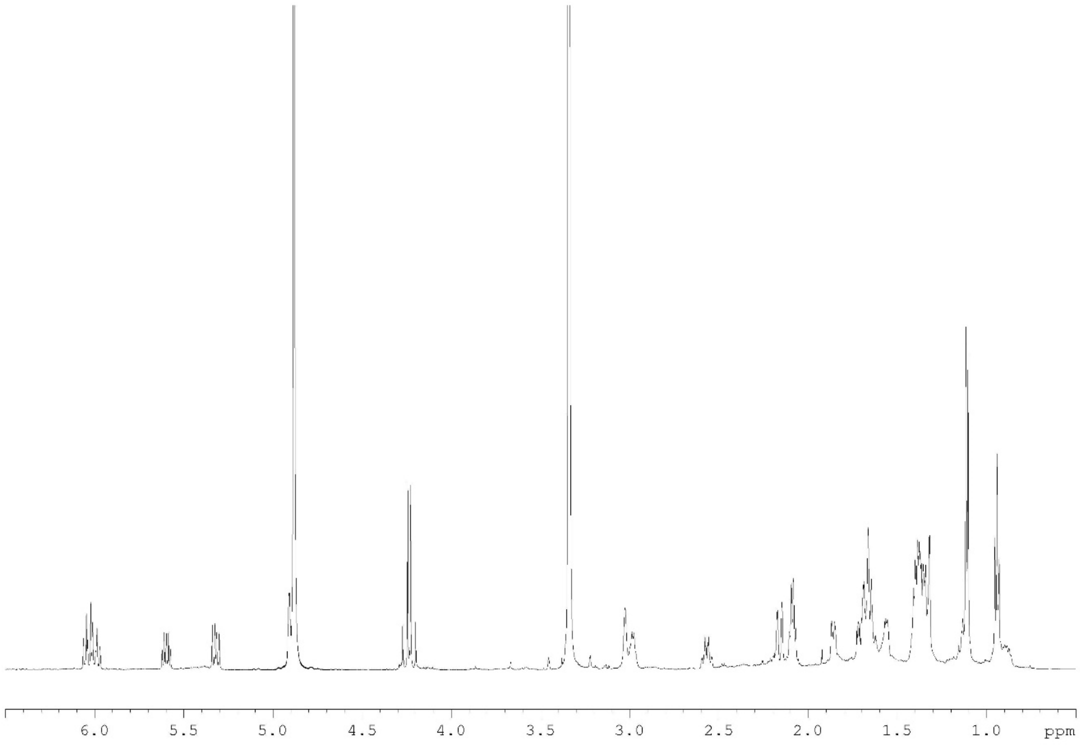

Figure S3. ^{1}H NMR (600 MHz, CD_3OD) spectrum of lepadin A from *C. lepadiformis* sp. B.

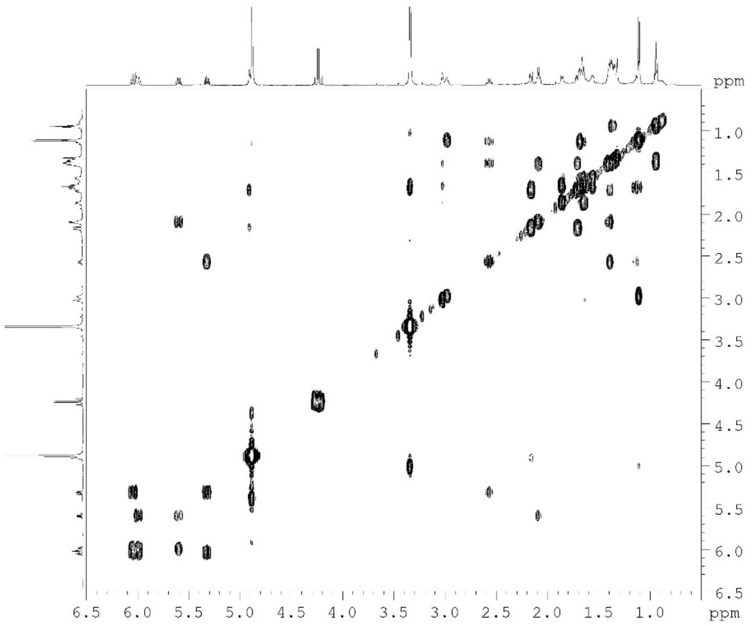

Figure S4. ^{1}H-^{1}H COSY NMR (600 MHz, CD_3OD) spectrum of lepadin A from *C. lepadiformis* sp. B.

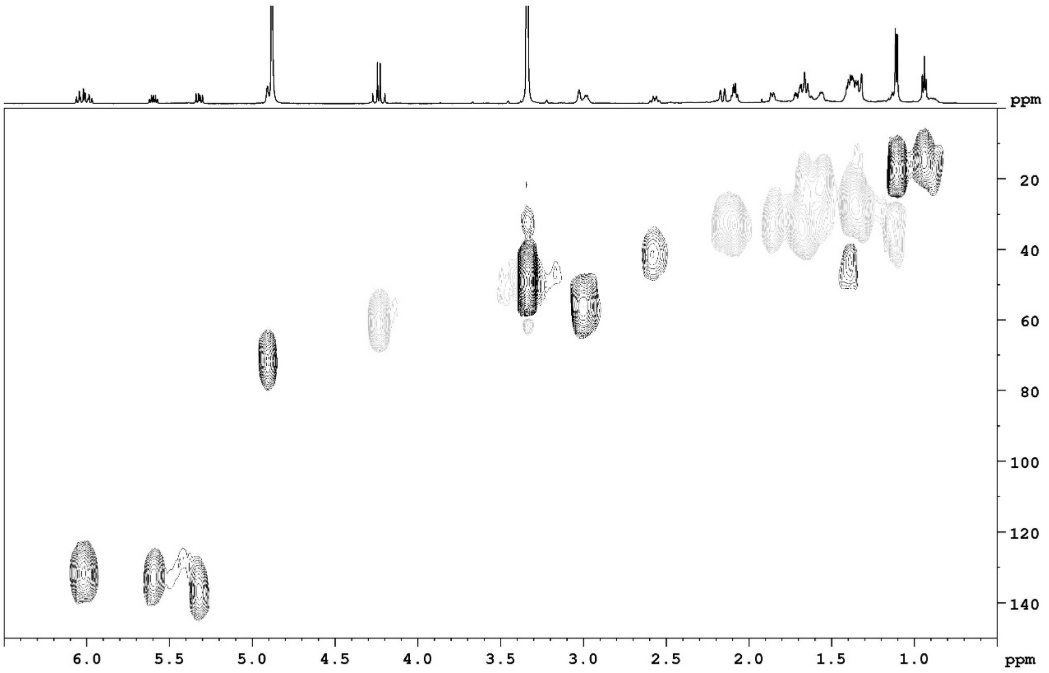

Figure S5. HSQCed NMR (600 MHz, CD_3OD) spectrum of lepadin A from *C. lepadiformis* sp. B.

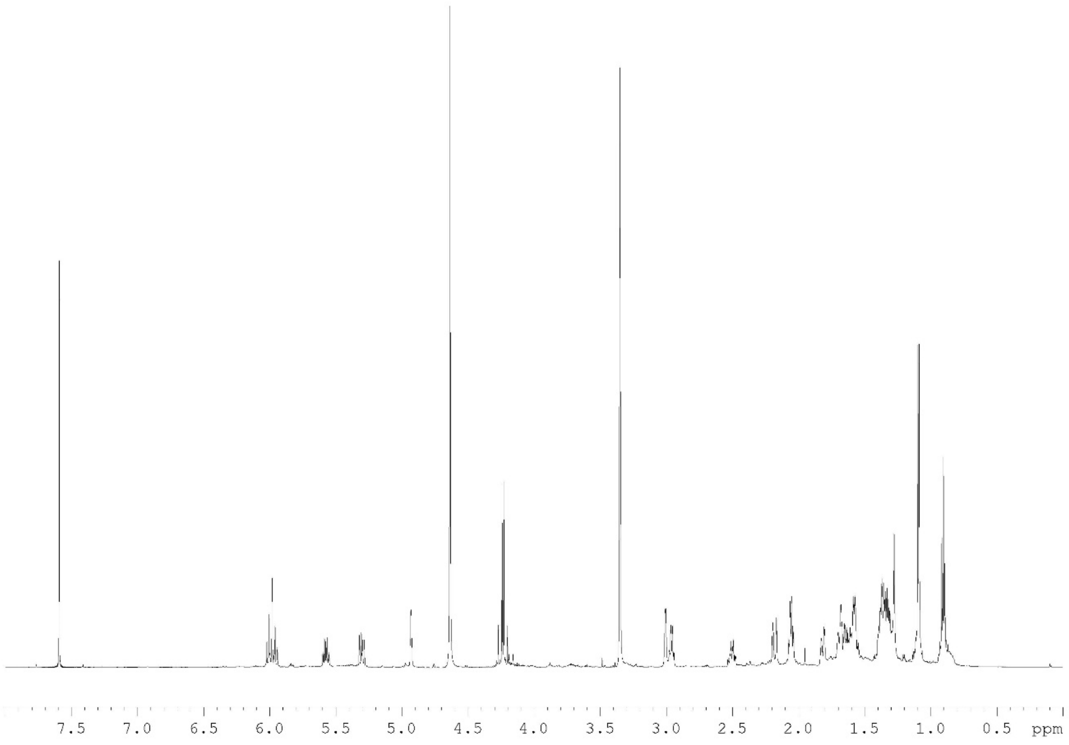

Figure S6. ^{1}H NMR (600 MHz, $CDCl_3$:CD_3OD 1:1 *v*/*v*) spectrum of lepadin A from *C. lepadiformis* sp. B.

Table S1. Blast percentage of similarity of "*Clavelina lepadiformis*" sequences from the Fusaro Lake (Bacoli, Tyrrhenian Sea, Mediterranean Sea) (sequences after Tarjuelo et al. [20], Turon et al. [18], Turon and López-Legentil [42], Gissi et al. [43], Reinhardt et al. [24], Stach et al. [44], Rius et al. [45], López-Legentil et al. [31], Holman et al. [46]).

Sample Code (GenBank)	Blast	GenBank (References)
**SZN-B-1046ASC15A SZN-B-1048ASC15C SZN-B-1049ASC15D SZN-B-1050ASC15E SZN-B-1051ASC15F (OM278387)**	98.1–100%	AF368352 (Tarjuelo et al. [20])<br>AM292603 (Gissi et al. [43])<br>AY211529–31 (Turon et al. [18])<br>AY603104 (Turon and López-Legentil [42])<br>FJ839918 (Stach et al. [44])<br>JX244863–4 (Turon et al. [18]; Rius et al. [45])<br>JX244871–2 (Turon et al. [18]; Rius et al. [45])<br>KF309563 (López-Legentil et al. [31])<br>MZ882307–11 (Holman et al. [46])<br>HM012482–3 (Reinhardt et al. [24])
	95.08–95.9%	JX244865–70 (Turon et al. [18] in Rius et al. [45])<br>KF309638 (López-Legentil et al. [31])<br>AF368353 (Tarjuelo et al. [20])

Table S2. GenBank COI partial sequences of *Clavelina* taxa (*C. lepadiformis* and *C. oblonga*) (after Blast results, Goddard-Dwyer et al. [47], and Rocha et al. [48]) and *Didemnum vexillum* (after Stefaniak et al. [49]) used in the phylogenetic analysis and associated specimen data (codes and clades/haplotypes after GenBank, Tarjuelo et al. [20], Turon et al. [18], and Rius et al. [45]; geographic localities obtained from GenBank and/or relevant paper/s). Abbreviations used: EAO—Eastern Atlantic Ocean; MED—Mediterranean Sea; PO—Pacific Ocean; SA—South Africa; WAO—Western Atlantic Ocean.

Taxon	GenBank	Code/Clade (h)	Locality	Reference
C. lepadiformis	AF368352	Atl+Interior (h 8)	MED, Estartit, Blanes, Port Ginesta	Tarjuelo et al. [20]
C. lepadiformis	AF368353	Exterior (h 1)	MED, Cadaques, Tossa, Blanes	Tarjuelo et al. [20]
C. lepadiformis	AY211529	Atl+Interior (h 9)	EAO, Vigo, Azores, Gullmarsfjorden; MED, Estartit, Blanes, Port Ginesta	Turon et al. [18]
C. lepadiformis	AY211530	Atl+Interior (h 12)	EAO, Sesimbra	Turon et al. [18]
C. lepadiformis	AY211531	Atl+Interior (h 13)	EAO, Azores	Turon et al. [18]
C. lepadiformis	JX244863	Atl+Interior (h 3)	SA	Turon et al. [18]; Rius et al. [45]
C. lepadiformis	JX244864	Atl+Interior (h 4)	EAO, Azores; SA	Turon et al. [18]; Rius et al. [45]
C. lepadiformis	JX244865	Exterior (h 2)	MED, Cadaques	Turon et al. [18] in Rius et al. [45]
C. lepadiformis	JX244866	Exterior (h 3)	MED, Tossa	Turon et al. [18] in Rius et al. [45]
C. lepadiformis	JX244867	Exterior (h 4)	MED, Tossa	Turon et al. [18] in Rius et al. [45]

C. lepadiformis	JX244868	Exterior (h 5)	MED, Blanes	Turon et al. [18] in Rius et al. [45]
C. lepadiformis	JX244869	Exterior (h 6)	MED, Blanes	Turon et al. [18] in Rius et al. [45]
C. lepadiformis	JX244870	Exterior (h 7)	MED, Blanes	Turon et al. [18] in Rius et al. [45]
C. lepadiformis	JX244871	Atl+Interior (h 10)	MED, Estartit; EAO, Azores; SA	Turon et al. [18]; Rius et al. [45]
C. lepadiformis	JX244872	Atl+Interior (h 11)	MED, Port Ginesta; EAO, Azores; SA	Turon et al. [18]; Rius et al. [45]
C. lepadiformis	KF309563	CF-4	MED, Spain	López-Legentil et al. [31]
C. lepadiformis	KF309638	ROS-CLY	MED, Spain	López-Legentil et al. [31]
C. lepadiformis	MZ882307	TB_H1	SA	Holman et al. [46]
C. lepadiformis	MZ882308	DB_H2	SA	Holman et al. [46]
C. lepadiformis	MZ882309	EL_H3	SA	Holman et al. [46]
C. lepadiformis	MZ882310	PE_H4	SA	Holman et al. [46]
C. lepadiformis	MZ882311	KN_H5	SA	Holman et al. [46]
C. lepadiformis	**OM278387**	**see Table S1**	**MED, Fusaro Lake**	**present paper**
C. oblonga	MK397830	HS36_08	WAO, Beaufort	Goddard-Dwyer et al. [47]
C. oblonga	JN859182	FL2	WAO, Florida	Rocha et al. [48]
D. vexillum	EU419401	--	PO, Japan	Stefaniak et al. [49]

Tables S1 and S2. References (numbers as in main text)

18. Turon, X.; Tarjuelo, I.; Duran, S.; Pascual, M. Characterising invasion processes with genetic data: An Atlantic clade of *Clavelina lepadiformis* (Ascidiacea) introduced into Mediterranean harbours. *Hydrobiologia* **2003**, *503*, 29–35.

20. Tarjuelo, I.; Posada, D.; Crandall, K.A.; Pascual, M.; Turon, X. Cryptic species of *Clavelina* (Ascidiacea) in two different habitats: Harbours and rocky littoral zones in the northwestern Mediterranean. *Mar. Biol.* **2001**, *139*, 455–462.

24. Reinhardt, J.F.; Stefaniak, L.M.; Hudson, D.M.; Mangiafico, J.; Gladych, R.; Whitlatch, R.B. First record of the non-native light bulb tunicate *Clavelina lepadiformis* (Müller, 1776) in the northwest Atlantic. *Aquat. Invasions* **2010**, *5*, 185–190.

31. López-Legentil, S.; Legentil, M.L.; Erwin, P.M.; Turon, X. Harbor networks as introduction gateways: Contrasting distribution patterns of native and introduced ascidians. *Biol. Invasions* **2015**, *17*, 1623–1638.

42. Turon, X.; López-Legentil, S. Ascidian molecular phylogeny inferred from mtDNA data with emphasis on the Aplousobranchiata. *Mol. Phylogenet. Evol*. **2004**, *33*, 309–320, doi:10.1016/j.ympev.2004.06.011.

43. Gissi, C.; Pesole, G.; Mastrototaro, F.; Iannelli, F.; Guida, V.; Griggio, F. Hypervariability of ascidian mitochondrial gene order: exposing the myth of deuterostome organelle genome stability. *Mol. Biol. Evol.* **2010**, 27, 211–215, doi: 10.1093/molbev/msp234.

44. Stach, T.; Braband, A.; Podsiadlowski, L. Erosion of phylogenetic signal in tunicate mitochondrial genomes on different levels of analysis. *Mol. Phylogenet. Evol.* **2010**, *55*, 860–870, doi:10.1016/j.ympev.2010.03.011.

45. Rius, M.; Clusella-Trullas, S.; McQuaid, C.D.; Navarro, R.A.; Griffiths, C.L.; Matthee, C.A.; von der Heyden, S.; Turon, X. Range expansions across ecoregions: interactions of climate change, physiology and genetic diversity. *Glob. Ecol. Biogeogr.* **2014**, *23*, 76–88, doi:10.1111/geb.12105.

46. Holman, L.E.; Parker-Nance, S.; de Bruyn, M.; Creer, S.; Carvalho, G.; Rius, M. Managing human-mediated range shifts: understanding spatial, temporal and genetic variation in marine non-native species. *Phil. Trans. R. Soc. B* **2022**, *377*, 20210025, doi:10.1098/rstb.2021.0025.

47. Goddard-Dwyer, M.; López-Legentil, S.; Erwin, P.M. Microbiome variability across the native and invasive ranges of the ascidian *Clavelina oblonga*. *Appl. Environ. Microbiol.* **2021**, *87*, e02233-20, doi:10.1128/AEM.02233-20.

48. Rocha, R.M.; Kremer, L.P.; Fehlauer-Ale, K.H. Lack of COI variation for *Clavelina oblonga* (Tunicata, Ascidiacea) in Brazil: Evidence for its human-mediated transportation? *Aquat. Invasions* **2012**, *7*, 419–424, doi:10.3391/AI.2012.7.3.012.

49. Stefaniak, L.; Lambert, G.; Gittenberger, A.; Zhang, H.; Lin, S.; Whitlatch, R.B. Genetic conspecificity of the worldwide populations of *Didemnum vexillum* Kott, 2002. *Aquat. Invasions* **2009**, *4*, 29–44, doi:10.3391/ai.2009.4.1.3.
